# Case report: Obstructive azoospermia as the first presentation of Von Hippel-Lindau disease

**DOI:** 10.3389/fonc.2023.1296555

**Published:** 2023-12-12

**Authors:** Raffaele Scafa, Maurizio Merico, Alfonso Massimiliano Ferrara, Sara Watutantrige Fernando, Pava Srsen, Francesca Schiavi, Stefania Zovato, Alberto Ferlin

**Affiliations:** ^1^ Department of Medicine, University of Padova, Padova, Italy; ^2^ Unit of Andrology and Reproductive Medicine, University Hospital of Padova, Padova, Italy; ^3^ Familial Cancer Unit, Veneto Institute of Oncology Istituto Oncologico Veneto (IOV)-IRCSS, Padova, Italy; ^4^ Department of Medicine, Unit of Surgical Pathology and Cytopathology, University Hospital of Padova, Padova, Italy

**Keywords:** azoospermia, male infertility, papillary cystadenoma of the epididymis, Von Hippel Lindau, VHL

## Abstract

We report the case of a 38-year-old man whose diagnostic workup for primary infertility led to the discovery of obstructive azoospermia due to bilateral papillary cystadenoma of the epididymis (PCE). Given the rarity of this finding and because PCE could be a manifestation of Von Hippel-Lindau disease (VHL), although the patient had no family or personal history of VHL, the *VHL* gene was tested, and a known pathogenetic variant (c.464-1G>A; p.)? was found. Screening for other Von Hippel-Lindau disease-associated neoplasms revealed bilateral retinal capillary hemangioblastomas, clear cell renal cell carcinoma, and multiple pancreatic cysts. In this case, an accurate diagnostic workup for male infertility allowed the detection of a rare life-threatening syndrome, already presenting with several silent neoplasms. For this reason, this case report may be useful for reproductive medicine specialists in the management of male infertility.

## Introduction

Von Hippel-Lindau disease (VHL) is an autosomal dominant syndrome that predisposes to the development of several malignant and benign neoplasms: retinal and central nervous system hemangioblastomas, clear cell renal cell carcinoma (ccRCC), pheochromocytomas and paragangliomas, pancreatic neuroendocrine tumors, pancreatic and renal cysts, endolymphatic sac tumors (ELST), epididymal cysts and cystadenomas in men, and cystadenomas of the broad ligament of the uterus in women (1). The estimated incidence of this condition ranges from 1 in 36,000 to 1 in 45,000 live births (2). Men and women are equally affected, with a mean age at tumor diagnosis of 26 years and an estimated penetrance of more than 90% by the age of 65 (3, 4).

VHL is caused by mutations in the *VHL* tumor suppressor gene (located at ch. 3p25.3), which encodes the VHL protein (pVHL) that regulates Hypoxia Inducible Factors (HIFs) (5). In normoxic conditions, pVHL binds to HIFs, leading to proteasome-mediated degradation. Under hypoxic conditions or loss of functional pVHL, HIF subunits are able to translocate to the nucleus and activate the transcription of many hypoxia-inducible genes (such as *VEGF*, *EPO*, *TGFα*, and *PDGFβ*), causing cell growth and angiogenesis (6). In 80% of cases, patients inherit a single germline mutant VHL allele from an affected parent, while in the other 20%, there is a *de novo* germline mutation. Tumor development occurs when the second wild-type copy is spontaneously lost or inactivated, according to Knudson’s two-hit hypothesis (3).

In individuals with a first-degree relative affected by VHL, the diagnosis of VHL is clinically established by the presence of a single typical VHL-related tumor (retinal or central nervous system (CNS) hemangioblastoma, or RCC). In the absence of a family history of VHL, the diagnosis requires two or more retinal or CNS hemangioblastomas, or one hemangioblastoma and one visceral tumor (excluding epididymal and renal cysts, which are common in the general population) (7).

Genetic testing can confirm or exclude a diagnosis in at-risk relatives from established families with VHL. Failure to find a disease-causing mutation in individuals with a suspected clinical diagnosis or individuals with an atypical presentation does not rule out a clinical diagnosis.

Despite the fact that VHL can cause manifestations at the epididymis level, such as cysts and cystadenomas, VHL screening is not included in the routine investigations for male infertility.

## Case description

A 38-year-old man was referred to our Unit of Andrology and Reproductive Medicine for primary infertility. Nothing relevant emerged from his family history. The patient denied any history of cryptorchidism, orchitis, epididymitis, or testicular trauma. The only relevant data was the detection at the age of 18 of bilateral epididymal cysts, which were not further investigated.

On physical examination, he had normotrophic testes (right 17 cc, left 14 cc) with voluminous, firm masses involving the heads of both epididymis, tender to palpation. The vas deferens were apparently normal. Physical examination revealed obesity (height 166 cm, weight 102 kg, BMI 37 kg/m^2^) and excluded gynecomastia, inguinal lymphadenopathies, and inguinal hernia. Two semen analyses showed azoospermia (no sperm observed after centrifugation) with normal volume (2.0 ml) and pH (7.2).

The endocrine assessment showed normogonadotropic hypogonadism (total testosterone 8.94 nmol/l, LH 4.7 IU/L, FSH 8.9 IU/L), and tumor markers (α-fetoprotein, β-hCG, and LDH isoenzymes) were negative.

Testicular ultrasound (US) showed bilateral epididymal head enlargement (right 33 mm and left 30 mm) due to lobulated cystic masses, which also showed increased vascularization on color Doppler US. Moreover, the US revealed signs of bilateral congestion of the rete testis. Transrectal ultrasound (TRUS) showed a slightly enlarged prostate (32 ml) with normoechoic texture, without focal lesions. The seminal vesicles and vas deferens were normal.

**Figure 1:**
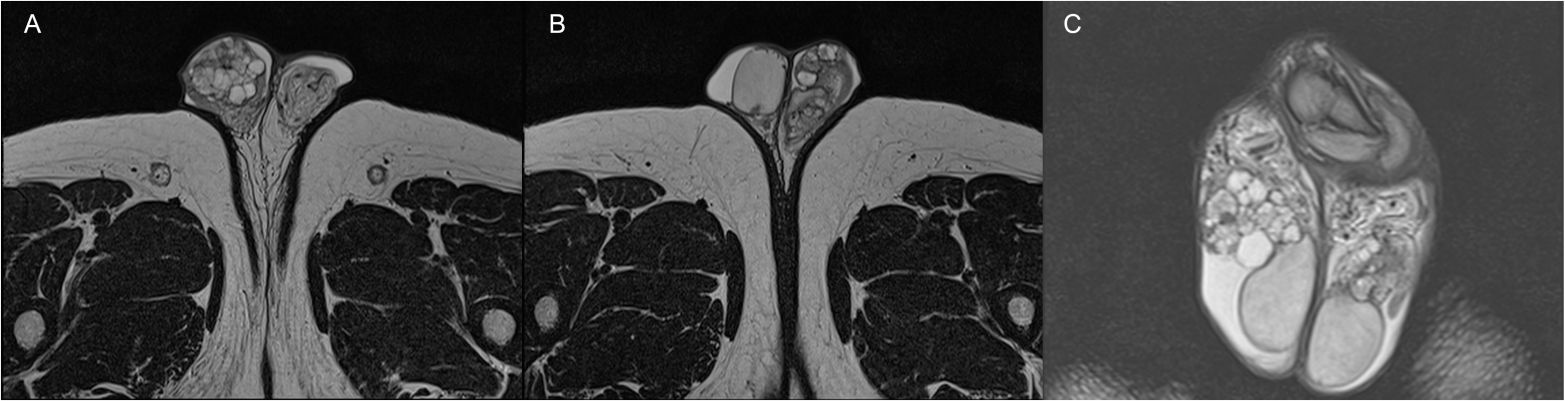
Axial **(A, B)** and coronal **(C)** TSE T2-weighted MRI images showing the presence of bilateral papillary cystadenoma of the epididymis.

For further evaluation, the patient underwent a scrotal and pelvic contrast-enhanced MRI, which described several cystic formations in both epididymis with non-homogeneous dense contents and proteinaceous aspects, consistent with the first hypothesis of bilateral spermatocele, according to the radiologist (**Figure 1**).

**Figure 2:**
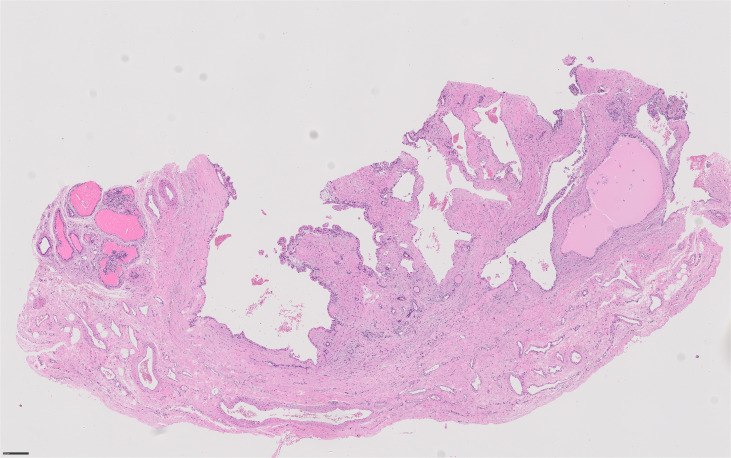
Histologic examination showing papillary cystadenoma of the epididymis (PCE).

We, therefore, performed bilateral testicular sperm extraction (TESE), which allowed the cryopreservation of sperm, and bilateral partial enucleation of epididymal cysts whose histologic examination showed bilateral papillary cystadenoma of the epididymis (PCE) (**Figure 2**).

**Figure 3:**
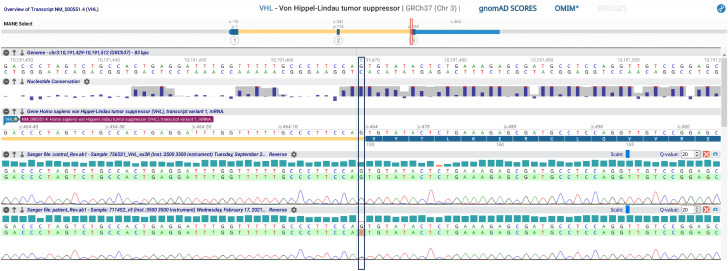
VHL gene sequencing analysis evidencing the variant c.464-1G>A.

Bilateral PCE is very rare and often pathognomonic for VHL. Consequently, the *VHL* gene was tested, and a known pathogenetic variant (c.464-1G>A; p.)? was found (**Figure 3**). Therefore, the patient underwent further evaluation in order to exclude the presence of other VHL-associated neoplasms. Fundoscopy revealed bilateral retinal capillary hemangioblastomas. Abdominal ultrasound showed multiple kidney cysts and three kidney masses highly suspicious for malignancy (two in the left kidney and one in the right kidney with peripheral calcific spots), which were enucleoresected, with histological examination confirming the diagnosis of ccRCC. The pancreatic structure was almost entirely subverted by numerous cysts up to 60 mm in diameter. Biochemical testing for pheochromocytoma by evaluation of 24-hour urinary fractionated metanephrines was negative.

## Discussion

Papillary cystadenoma of the epididymis (PCE) is the second most common benign neoplasm of the epididymis after adenomatoid tumors (8). It mostly affects young adults and may occur sporadically or in association with VHL (9). More than one-third of the PCE cases reported in the literature have occurred in VHL patients; this correlation is stronger in the case of bilateral tumor presentation (10, 11).

The diagnosis is primarily incidental because PCE is often asymptomatic. In symptomatic patients, the most common presentation is a painless, slowly growing scrotal swelling. Patients rarely complain of pain or tenderness in the scrotum. More rarely, when bilateral, as in our case, PCE can be a cause of infertility due to obstructive azoospermia. The prevalence of PCE in infertile patients is unknown.

To the best of our knowledge, this is the second case in which VHL was diagnosed due to primary infertility in an otherwise asymptomatic patient with no family history of VHL. The diagnostic work-up for infertility led to the identification of obstructive azoospermia due to bilateral PCE, which then allowed the detection of VHL, a life-threatening syndrome already associated with several silent neoplasms. Exams also led to the diagnosis of functional normogonadotropic hypogonadism related to obesity, after the exclusion of organic causes (12). Previously, de Souza Andrade et al. (13) described a patient who underwent surgical excision of a cerebellar hemangioblastoma and received a VHL diagnosis only 2 years later, when he was found to have bilateral PCE associated with obstructive azoospermia (14).

Regarding the infertility problem in this specific setting, standard approaches to obstructive azoospermia, which include sperm retrieval via percutaneous or open biopsy of the testes, are the preferred management strategies (15). Symptomatic PCE, instead, when feasible, is usually treated with testicle-sparing surgical excision.

## Conclusion

This case report may be of value to specialists in reproductive medicine in the management of male infertility; in fact, clinicians should be aware of the possibility that men with obstructive azoospermia may have bilateral PCE related to VHL disease. On the other hand, VHL should always be considered in patients with PCE, even in asymptomatic individuals with no family history of VHL, since they may be at risk for developing other VHL-associated tumors. This is especially true in the case of bilateral PCE, a condition that is almost pathognomonic for VHL (9).

Once a VHL gene mutation is identified in a proband, genetic testing should be offered to all first-degree relatives of the individual, even if the family history is negative, because reduced penetrance, later age of onset, variable expressivity among family members, or death of an affected parent before the onset of symptoms are reported in VHL (16).

Although preventive treatment cannot yet be offered for VHL, patients are recommended to undergo regular surveillance to ensure early diagnosis and timely treatment to prevent avoidable morbidity and mortality (17).

In the patient in this case report, the diagnosis of VHL allowed the early identification of malignant tumors that would otherwise have been detected much later and with a significantly worse prognosis.

In conclusion, VHL should always be considered in patients with papillary cystadenoma of the epididymis, especially when bilateral.

## Data availability statement

The raw data supporting the conclusions of this article will be made available by the authors, without undue reservation.

## Ethics statement

Ethical approval was not required for the studies involving humans because standard clinical care and case report for a subject who agreed to collect and publish his data anonimously. The studies were conducted in accordance with the local legislation and institutional requirements. The participants provided their written informed consent to participate in this study. Written informed consent was obtained from the individual(s) for the publication of any potentially identifiable images or data included in this article.

## Author contributions

RS: Data curation, Writing – review & editing, Conceptualization, Formal Analysis, Investigation, Writing – original draft. MM: Conceptualization, Investigation, Writing – original draft, Writing – review & editing. AMF: Investigation, Writing – review & editing, Data curation, Formal Analysis. SW: Data curation, Investigation, Writing – review & editing. PS: Investigation, Writing – review & editing. FS: Formal Analysis, Writing – review & editing. SZ: Data curation, Writing – review & editing, Supervision, Validation. AF: Data curation, Supervision, Validation, Writing – review & editing.
